# The Hypoglycemic and Hypolipidemic Effects of Polyphenol-Rich Strawberry Juice on Diabetic Rats

**DOI:** 10.1007/s11130-023-01079-1

**Published:** 2023-07-18

**Authors:** Hanaa S.S. Gazwi, Mohamed SH. Hassan, Hamadi A. Ismail, Gamal F. Abd El-Naem, Sallam K. Tony

**Affiliations:** Department of Agricultural Chemistry, Faculty of Agriculture, Minia University, El-Minia, Egypt

**Keywords:** Strawberry Juice, Diabetes, HPLC, Hyperlipidemia, Oxidative stress

## Abstract

Obesity and diabetes are some of the most important modern health problems requiring simple preventative or palliative measures using dietary means. This study investigated the impact of strawberry juice on diabetic rats. Diabetes was induced in rats using a single intraperitoneal injection of 50 mg/kg streptozotocin (STZ). Fifty male rats were divided into five groups: normal control (NC), strawberry juice only (S), diabetic control (DC), and two diabetic groups treated with strawberry juice (DC + S) or metformin (DC + met). Rats were administered a single dose of both strawberry juice and oral metformin, and biochemical and histological analyses were conducted. The experiment was conducted in compliance with the Ethics Committee’s regulations for the care and utilization of animals, microorganisms, and living cell cultures in education and scientific research at the Faculty of Agriculture, Minia University (MU/FA/006/12/22). Treatment of diabetic rats with strawberry juice led to a significant decrease in blood glucose. Insulin levels were also significantly increased, while lipid profiles were lowered in the diabetic rats treated with strawberry juice. Carbohydrate metabolism enzymes and antioxidant enzyme activities in the treated rats were restored to normal levels, and the levels of lipid peroxidation and proinflammatory cytokines were notably reduced. The microstructure of pancreatic and liver cells in diabetic rats was also improved with strawberry juice treatment. In addition, HPLC analysis revealed that strawberry juice was rich in flavonoids and phenolic compounds and exhibited potent antioxidant activity. These findings suggest that strawberry juice has considerable hypoglycemic and hypolipidemic effects on rats with diabetes which may be used in human after further investigations.

**Supplementary Information:**

The online version contains supplementary material available at 10.1007/s11130-023-01079-1.

## Introduction

Diabetes mellitus (DM), a persistent metabolic disorder, is characterized by insufficient insulin secretion from the pancreas and/or resistance to insulin in peripheral tissues. These conditions contribute to elevated levels of glucose in the bloodstream, a condition referred to as hyperglycemia. Streptozotocin (STZ) is used in experiments to induce a diabetic state as it selectively destroys pancreatic *β*-cells, resulting in hyperglycemia and insulin impairment [1]. Hyperinsulinemia and hyperglycemia can cause severe damage to organ function and joint complications [2]. Additionally, hyperglycemia is linked to the generation of reactive oxygen species (ROS), which can cause oxidative harm to the liver, pancreas, and kidneys [3].

Despite the availability of several antihyperglycemic medications derived from synthetic or natural sources, diabetes and its consequences remain major problems for which modern medicine has yet to produce a suitable solution [4]. The high cost and harmful effects of these agents, coupled with their limited efficacy, have created a pressing need for alternative treatments [1]. The World Health Organization (WHO) has recommended expanding the use of traditional herbal remedies for people with diabetes [5]. Medicinal plants have also been identified as a potentially useful source for the development of anti-diabetic medicines [6]. Among the most extensively researched substances for treating diabetes and its complications are phenolic compounds with positive effects and pharmacological properties [7].

Strawberries, scientifically known as *Fragaria x ananassa*, are a highly valuable fruit commercially and economically, with popular forms including juices, jams, and jellies. Strawberries are also a healthy food choice due to their high content of phytochemicals, such as minerals, folate, vitamins, fiber, and a mixture of polyphenols including phenolic acids, flavonoids, ellagitannins, anthocyanins, and tannins [8]. These phytochemicals are responsible for the antioxidant properties of strawberries, which contribute to the health benefits of consuming this fruit [9]. Research has documented the antioxidant properties of strawberries in both in vivo and in vitro settings [10]. Strawberries have been used for medicinal and nutritional purposes for a long time, and they provide a natural source of protection against harmful substances and diseases [8]. Consuming strawberries has been linked to a lower incidence of various chronic conditions, such as infections, obesity, cancer, and neurological and cardiovascular diseases [11].

The current study was designed to assess the potential ameliorative impact of strawberry juice on streptozotocin-induced diabetic rats.

## Materials and Methods

The material and methods section is presented as supplementary material.

## Results and Discussion

### Strawberry Juice Contains Bioactive Compounds and Antioxidants

The phytochemical screening of strawberry juices revealed the presence of numerous phenolic compounds. The total phenolic content (TPC) and total flavonoid content (TFC) of the strawberry juices were quantitatively assessed and found to be 28.8 ± 1.29 mg GAE g^− 1^ sample and 22.03 ± 1.74 mg g^− 1^ sample, respectively (Table S1). These results agree with the findings reported by Sreekumar et al. [12], who stated that all types of strawberries have a high phenolic content. These compounds play a crucial role in protecting the body against harmful free radicals [13, 14].

The antioxidant activity of strawberry juice was measured using different assays. The juice showed higher radical scavenging activity using the DPPH assay (25.22 ± 1.0 *µ*mol TE/mL) (Table S1). Moreover, strawberry juice exhibited high activity in the FRAP assay (47.59 ± 3.14 *µ*mol TE/mL) and the ABTS assay (144.69 ± 13.44 *µ*mol TE/mL) (Table S1). These findings are consistent with the results of previous studies conducted by Parikh and Patel [15], which demonstrated a significant correlation between phenolic content and antioxidant activity measured by DPPH, FRAP, and ABTS assays.

Table S2 displays the results of the analysis of the polyphenolic composition of strawberry juice. The chemical profile of the juice, as shown in Table S2 and Figure S1, consisted of seven flavonoid compounds and six phenolic compounds that were identified based on the retention time of standard compounds. The most abundant phenolic compound identified was catechol, followed by syringenic. Similarly, the most abundant flavonoid compound was rutin, followed by quercetin.

### Effects of Strawberry Juice on Body Weight in Diabetic Rats

The study found that the DC group had a significantly lower body weight compared to the NC group (*p* < 0.05), indicating weight loss likely due to muscle atrophy and accelerated breakdown of tissue proteins caused by STZ induction (Figure S2). Lower body weight in diabetic rats may be attributed to decreased insulin utilization and protein degradation resulting from the inability to use carbohydrates as an energy source, as suggested by Kanchana et al. [16]. However, diabetic rats treated with strawberry juice or metformin experienced a significant increase in final body weight compared to the DC group (*p* < 0.05), as shown in Figure S2. Oral intake of strawberry juice helped maintain protein turnover and improve glycemic control, leading to the restoration of body weight.

### Effects of Strawberry Juice on Liver and Kidney Functions

**Fig. 1:**
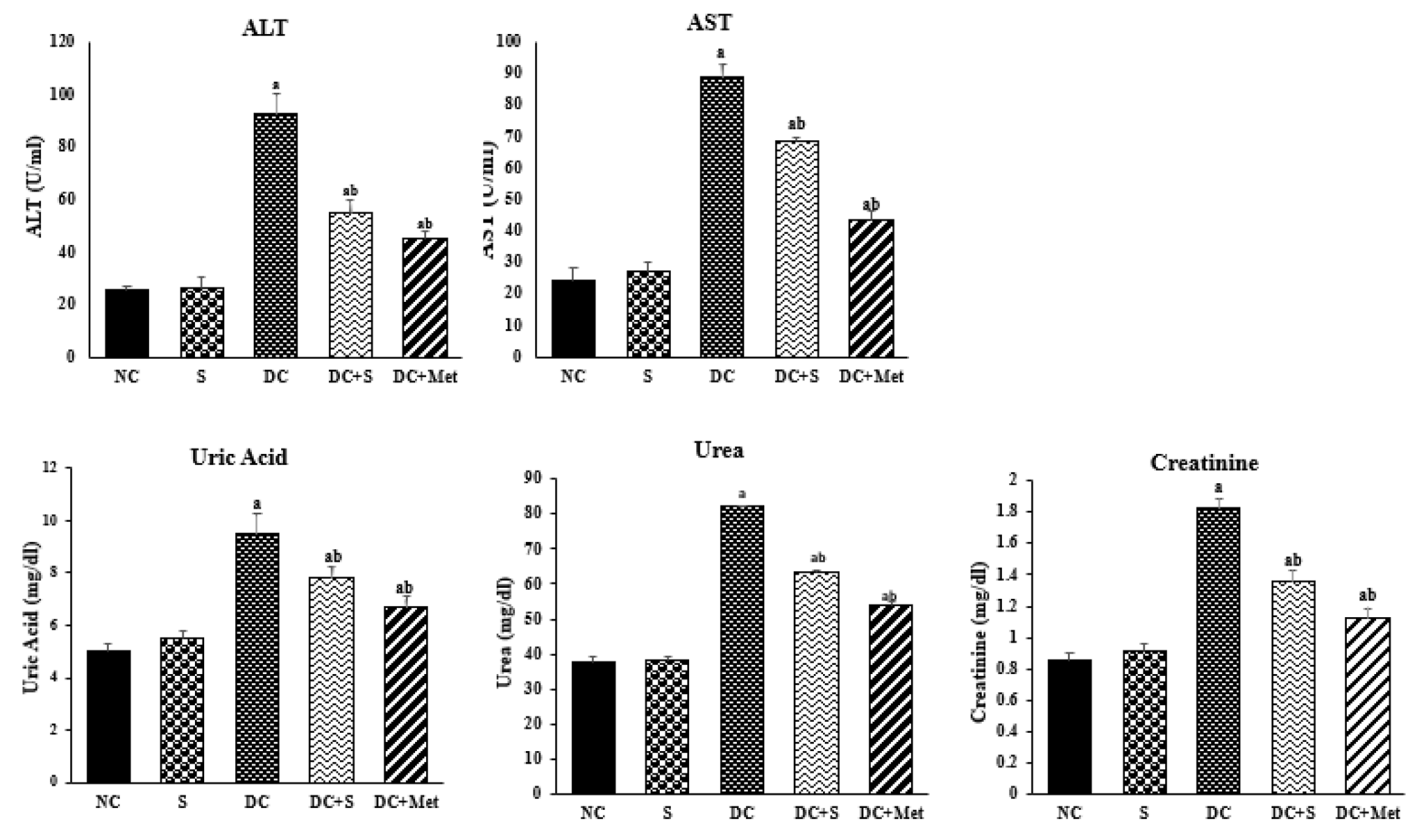
Effects of strawberry juice on liver and kidney functions (means ± SE). ^a^ and ^b^ are significant at *p* < 0.05 in comparison of groups with normal and diabetic control groups, respectively. NC, normal control; S, strawberry juice; DC, diabetic control; Met, metformin

Figure 1 summarizes the effects of strawberry juice on liver and kidney profiles. The DC group had significantly higher levels of AST and ALT in the liver compared to the NC group (*p* < 0.05), indicating liver damage induced by STZ. Strawberry juice treatment reduced AST and ALT levels to near-normal levels, similar to the effects of metformin. The decrease in ALT activity may be attributed to the influx of antioxidants from strawberries into the liver. In terms of kidney function, the DC group exhibited increased levels of creatinine, uric acid, and urea compared to the NC group, indicating renal damage (Fig. 1). However, administering strawberry juice to diabetic rats resulted in a notable decline in these kidney function indicators, suggesting the potential efficacy of the juice in mitigating kidney impairment. The improvement in kidney function was less significant with strawberry juice compared to metformin. These findings align with Murillo-Villicaña et al. [17], who reported STZ-induced renal impairment in rats characterized by elevated levels of uric acid, urea, and creatinine.

### Effects of Strawberry Juice on Lipid Profile

**Fig. 2:**
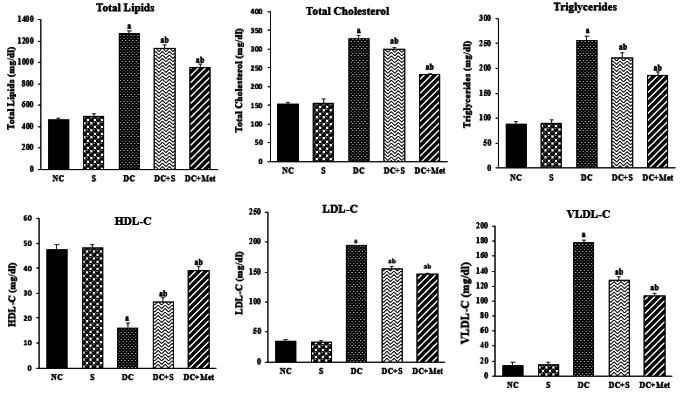
Effects of strawberry juice on lipid profile (means ± SE). ^a^ and ^b^ are significant at *p* < 0.05 in comparison of groups with normal and diabetic control groups, respectively. NC, normal control; S, strawberry juice; DC, diabetic control; Met, metformin

Figure 2 depicts the results of the study’s analysis on the effects of strawberry juice on the lipid profile. The diabetic rats (DC group) exhibited significantly lower HDL-C levels compared to the normal control (NC) group (*p* < 0.05). However, administering strawberry juice to diabetic rats significantly increased HDL-C levels, similar to the medication-treated group. Conversely, the DC group demonstrated significantly higher LDL-C, triglyceride, and VLDL-C levels than the NC group (*p* < 0.05), as illustrated in Fig. 2. Administering strawberry juice alleviated these levels in diabetic rats. These findings are consistent with Murillo-Villicaña et al. [17] and emphasize the intricate relationship between hyperglycemia and dyslipidemia in diabetes, underscoring the importance of addressing both for disease management.

The lipid-lowering effects of flavonoids in fruit juices, including quercetin, align with other published findings [18]. These effects can be attributed to various mechanisms. Firstly, flavonoids may inhibit cholesterol absorption and enhance the catabolism of triglyceride-rich lipoproteins. Additionally, they can increase bile flow and promote the excretion of bile cholesterol and bile acids, as demonstrated for naringin [19]. Flavonoids can also inhibit the enzyme 3-hydroxy-3-methylglutaryl-CoA reductase, thereby reducing cholesterol synthesis. Furthermore, they can inhibit the enzyme acyl-CoA:cholesterol acyltransferase, resulting in decreased cholesterol esterification in the intestine and liver. Consequently, this leads to reduced cholesterol absorption and its incorporation into lipoproteins. These inhibitory activities have been observed for various flavonoids, including quercetin [19], which is a constituent of the juice.

### Effects of Treatment with Strawberry Juice on Levels of Insulin, Glucose, and Carbohydrate Metabolizing Enzymes

**Fig. 3:**
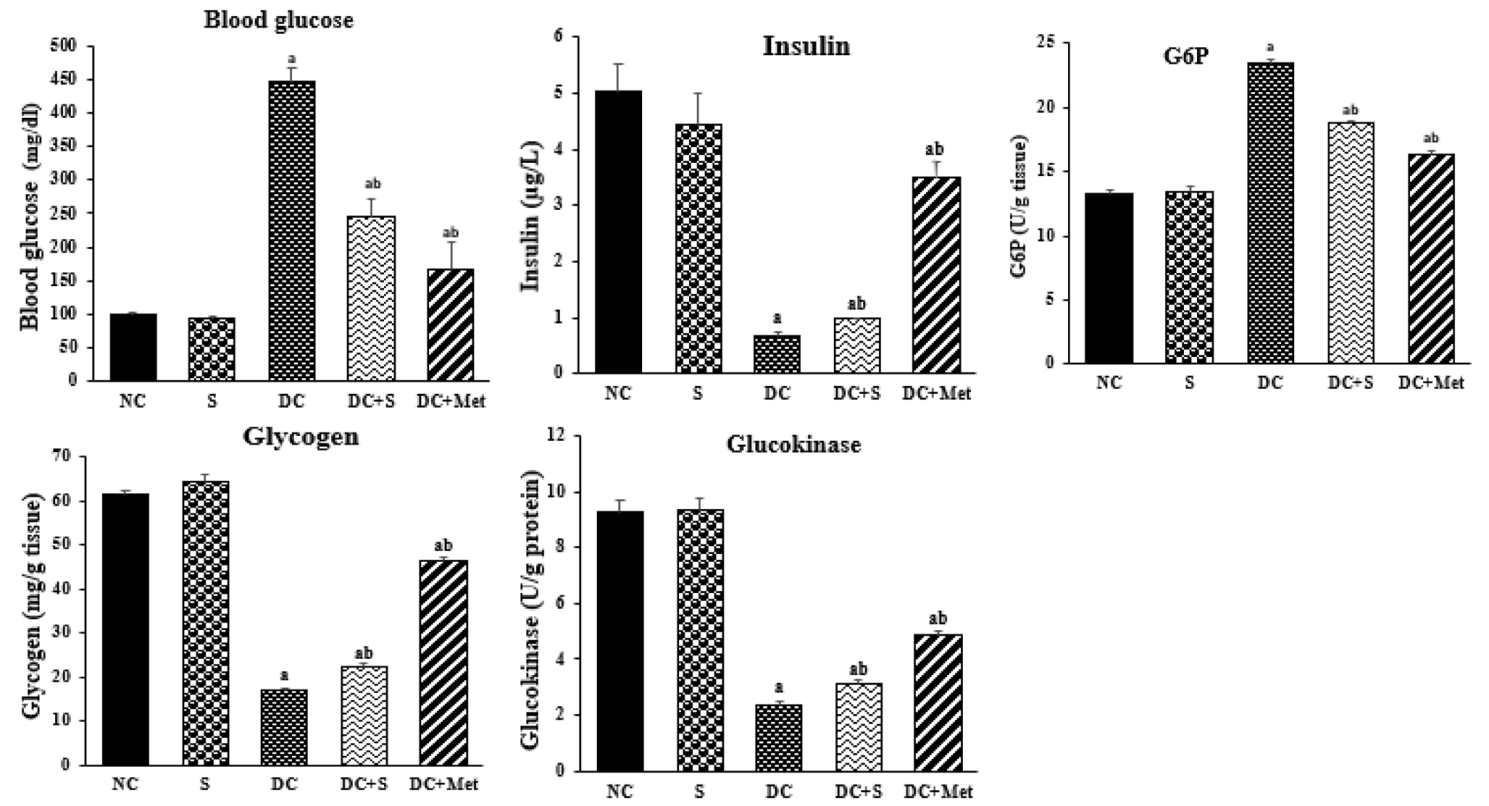
Effects of strawberry juice on levels of insulin, glucose, and carbohydrate metabolizing enzymes (means ± SE). ^a^ and ^b^ are significant at *p* < 0.05 in comparison with groups with normal and diabetic control groups, respectively. NC, normal control; S, strawberry juice; DC, diabetic control; Met, metformin

Figure 3 shows that the diabetic control (DC) group had decreased insulin levels and increased glucose levels compared to the normal control (NC) group. However, administering strawberry juice to diabetic rats significantly improved insulin and glucose levels compared to the DC group (*p* < 0.05). This aligns with Murillo-Villicaña et al. [17], who found that STZ increased glucose levels, indicating diabetes induction. Strawberry juice consumption, as reported by Kanchana et al. [16], may be a potential dietary intervention for managing diabetes by improving glucose and insulin levels. Strawberry polyphenols may promote insulin secretion and protect *β*-cells from STZ-induced oxidative stress. Additionally, strawberry juice contains significant amounts of phenolic compounds, particularly caffeic acid, which has the potential to suppress the rise in blood glucose levels by inhibiting the activity of the *α*-glucosidase enzyme in the small intestine [20]. Consequently, strawberry juice could serve as a viable alternative for the treatment of diabetes mellitus (DM).

Figure 3 also demonstrates changes in carbohydrate-metabolizing enzymes, with diabetic rats showing reduced activities of glucose kinase and glycogen, and increased glucose-6-phosphatase (G6P) compared to normal rats (*p* < 0.05). Administration of strawberry juice or metformin to diabetic rats reversed these changes in enzyme activity, with significant improvements (*p* < 0.05) observed in rats treated with strawberry juice. Mandave et al. [21] supported these findings, suggesting that strawberry extract boosts insulin levels, enhances insulin sensitivity, and promotes glucose regulation in diabetic rats by regulating IL-6 and promoting *β*-cell regeneration/protection.

### Effects of Strawberry Juice on Oxidative Stress Parameters and Inflammatory Markers

**Fig. 4:**
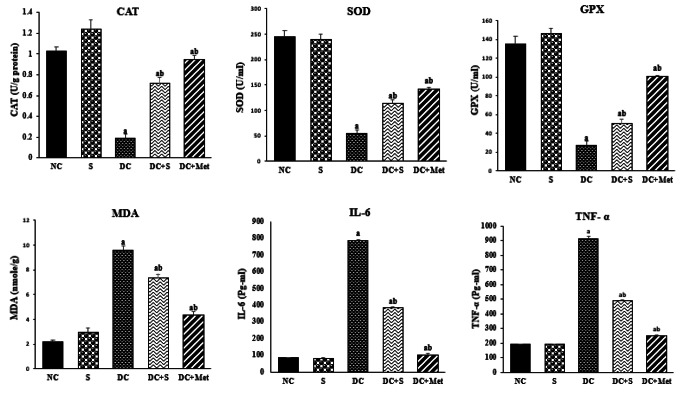
Effects of strawberry juice on oxidative stress and inflammatory markers (means ± SE). ^a^ and ^b^ are significant at *p* < 0.05 in comparison with groups with normal and diabetic control groups, respectively. NC, normal control; S, strawberry juice; DC, diabetic control; Met, metformin

According to Fig. 4, the DC group had lower levels of GSH, SOD, and CAT compared to the NC group. However, strawberry juice significantly increased these parameters in diabetic rats (*p* < 0.05). Additionally, MDA levels, indicating lipid peroxidation, were higher in the DC group but were effectively restored by strawberry juice (*p* < 0.05). According to Kanchana et al. [16], the development and complications of diabetes result from an imbalance between ROS production and the ROS scavenging system. In diabetes, ROS can arise due to protein glycation and glucose autoxidation, leading to lipid peroxidation [22]. Oxidative stress can impair the structural integrity of pancreatic *β*-cells, causing endocrine dysfunction [22]. Murillo-Villicaña et al. [17] have reported that diabetic rats exhibit an increase in lipid peroxidation markers such as H_2_O_2_ and TBARS and a decrease in antioxidant enzymes such as SOD, GPX, and CAT. Likewise, Zhou et al. [23] found that STZ-induced diabetic rats showed a significant rise in MDA levels, indicating an increase in lipid peroxidation and oxidative stress, as well as a significant decrease in SOD levels, indicating a reduction in antioxidant capacity.

Antioxidants protect against cellular damage caused by ROS, and phenolic compounds play a significant role in this protection. Regular consumption of these compounds in the diet is important since the human body does not naturally produce them. Strawberry juice’s antioxidant properties reduce oxidative stress, improve pancreatic *β*-cell function, and enhance insulin production and secretion.

Strawberry juice also exhibits anti-inflammatory properties, as seen in Fig. 4 with significantly reduced levels of IL-6 and TNF-*α* compared to the DC group (*p* < 0.05). Chronic inflammation and inflammatory cytokines contribute to the pathophysiology of diabetes, particularly Type 1 Diabetes Mellitus, by damaging pancreatic *β*-cells [24]. Strawberry juice’s anti-inflammatory qualities may help mitigate the effects of chronic inflammation and reduce the risk of diabetes complications.

### Histopathological Examination of Liver and Pancreas

Histological examination of hepatic tissue in the NC and S groups showed normal cells without any abnormalities under the microscope (Figures S3 A-B and Table S3). In contrast, diabetic rats exhibited Kupffer cell activation, sporadic cell necrosis, vacuolar degeneration, and portal edema in the liver tissue (black arrows) (Figure S4 C and Table S3). Treatment with strawberry juice in the DC + S group improved hepatic tissue, with slight Kupffer cell activation, some necrosis, and cavities in hepatocytes (black arrows) (Figure S4 D and Table S3). Similarly, histopathology of the liver tissue in the DC + Met group showed minor vacuoles in some hepatocytes (black arrows) (Figure S4 E and Table S3) in a few sections.

Pancreatic histology examination in rats from the normal control and strawberry groups revealed normal islets of Langerhans and pancreatic acini (Figure S4 A-B and Table S4). Diabetic rats exhibited necrosis and emptying of Langerhans cells in the pancreas (black arrows), along with significant enlargement of the epithelium lining the pancreatic duct and thickening of its wall (black arrows) (Figure S4 C-D and Table S4). The pancreas of the DC + S group showed no histopathological changes, except for slight congestion in pancreatic blood vessels in some sections (black arrows) (Figure S4 E and Table S4). The pancreas of diabetic rats treated with metformin showed no histopathological changes (black arrows) (Figure S4 F and Table S4).

Liver damage in diabetic patients is primarily caused by hyperglycemia-induced oxidative stress [25]. Histopathological analysis of pancreas, liver, and kidney sections has shown disturbances in morphological features in these organs, as noted by Ahmed et al. [26]. According to Mohamed et al. [25], the observed histological changes in the pancreas of diabetic rats were caused by reactive oxygen species (ROS). Vacuolation, considered an indicator of membrane permeability disturbances, can be caused by ROS through the formation of lipid peroxides, as discussed by Mohamed et al. [25].

After 56 days of treatment with strawberry juice, STZ-induced diabetic rats showed nearly normal beta-cell-containing islets of Langerhans. The central vein and hepatocytes in the liver were also restored to their normal function. The natural morphology of the descending and ascending loops was almost completely restored. Insulin, glucose, and carbohydrate metabolizing enzymes returned to normal levels, providing strong evidence for the reversal of histopathological changes. Mandave et al. [21] demonstrated that strawberry extract protected *β*-cells, reduced pancreatic tissue damage, and controlled/prevented liver tissue damage in rats, as observed through histological examination of pancreatic cells.

## Conclusion

Our findings indicate that strawberry juice can reduce blood glucose levels by stimulating pancreatic *β*-cells to produce more insulin. Furthermore, strawberry juice can decrease inflammation and oxidative stress by reducing inflammatory indicators such as IL-6 and TNF-*α* while increasing antioxidant enzyme activity. These findings are supported by histopathological studies. Therefore, we propose that strawberry juice can be used as a functional food due to its high concentration of natural antioxidants, which may help prevent diabetes and hypercholesterolemia.

## Electronic Supplementary Material

Below is the link to the electronic supplementary material.

Supplementary Material 1

## Data Availability

The datasets utilized and analyzed during this investigation are available upon reasonable request from the corresponding author.
