# Supplementary material for: The Hypoglycemic and Hypolipidemic Effects of Polyphenol-Rich Strawberry Juice on Diabetic Rats
Source: Plant Foods Hum Nutr. 2023 Jul 18;78(3):512–9. doi: 10.1007/s11130-023-01079-1 (PMC10495482; doi:10.1007/s11130-023-01079-1)
Supplement: Supplementary file 1 — Supplementary Material 1 [file 11130_2023_1079_MOESM1_ESM.docx]

**Materials and methods**

**Preparation of strawberry fruit juice**

Strawberry fruits (*Fragaria ananassa*) were carefully washed and had their leaves removed. They were then sliced into pieces using a stainless-steel knife. To prepare the fruit juice, a commercial blender (Philips, China) was used to blend the strawberry pieces, and the resulting juice was immediately diluted with distilled water to a 1:1 volume ratio. The juice was then filtered to remove any solid pieces, resulting in a clear liquid. Finally, the clear juice was stored at a temperature of -20 °C until for used.

**Measurement of Bioactive compounds**

The strawberry juice was filtered using Whatman No. 1 filter paper and subsequently evaporated using a rotary evaporator under reduced pressure at 50°C. The resulting sample was then used for the quantification of bioactive compounds in the strawberry juice.

**Measurement of total flavonoid content (TFC) and total phenolic contents (TPC)**

The Folin-Ciocalteu test [1] was employed to ascertain the total phenolic content (TPC), which was quantified as the quantity of gallic acid equivalent present in every 100 grams of dry material. Meanwhile, the total flavonoid content (TFC) was determined using the method delineated in [1] and reported as quercetin equivalent per 100 grams of dry material.

***In vitro* antioxidants activities**

The antioxidant capacity of strawberry sample was assessed using the ferric reducing antioxidant power (FRAP) assay, following the protocol described by Benzie and Strain [2]. Additionally, the radical scavenging activity of the sample against the stable DPPH radical was measured utilizing the method established by Saija et al. [3], while the sample's ability to scavenge the ABTS radical was measured according to the procedure outlined by Kutlu et al. [4].

**HPLC analysis**

The flavonoid and phenolic components of the sample were analyzed using the HPLC system, following the procedure described by Gazwi et al. [5]. A sample volume of 25 μL was injected into the Agilent 1100 HPLC system. To identify the phenolic components, a C18 column was used with a UV/Vis detector set at 250 nm. The solvents used were methanol and acetic acid in water (1:25) in a gradient program. The program started with 100% solvent B and maintained this for the first 3 minutes, followed by 5 minutes of 50% solvent A, 2 minutes of 80% solvent A, and 5 minutes of 50% solvent A. A detecting wavelength of 250 nm was selected. To determine the flavonoid compounds, present, the same HPLC system was used, this time with a C18 column (250 × 4.6 mm, 5 μm) and a UV/Vis detector set to 360 nm. The mobile phase consisted of acetonitrile (A) and 0.2% (v/v) aqueous formic acid (B), which were eluted in an isocratic 70:30 ratio.

**Experimental animals**

Fifty adult male albino Wistar rats, with an average weight of 150 ± 20 g and aged between 6 to 8 weeks, were procured from the Animal House at the Faculty of Pharmacy, Al-Nahda University, Beni Suef. These rats were maintained under standard light and dark cycles (21 ± 2 °C) and provided with food and water ad libitum in accordance with established standards. The experiment was conducted in compliance with the Ethics Committee's regulations for the care and utilization of animals, microorganisms, and living cell cultures in education and scientific research at the Faculty of Agriculture, Minia University (MU/FA/006/12/22).

**Induction of experimental diabetes**

To induce diabetes, rats were administered an intraperitoneal injection of STZ (50 mg/kg body weight) dissolved in a 0.1 M citrate buffer with pH 4.5. After 72 hours, their fasting blood glucose levels were assayed using a blood glucose meter from the tail vein. Consistent with the observations of Samarghandian et al. [6], rats were included in the study if their blood glucose levels were 250 mg/dL or higher.

**Experimental design**

The study involved 50 rats, which were divided into five groups randomly, each group containing 10 rats.

**Normal Control (NC) group:** Normal control rats

**Strawberry juice (S) group:** Normal rats were given strawberry juice (9 mL/kg b. wt) by oral tube daily for 56 days [7].

**Diabetic Control (DC) group:** Diabetic Control rats

**Diabetic + Strawberry Juices (DC+ S) group:** Diabetic rats were given strawberry juice (9 mL/kg b. wt) by oral tube daily for 56 days

**Diabetic + Metformin (DC+ M) group:** Diabetic rats were given metformin (500 mg /kg b. wt) by oral tube daily for 56 days [8].

At the end of the 56-day experiment, the rats underwent a 12-hour fast, and their fasting blood glucose levels were determined by collecting a blood sample from the tail vein using a glucometer. Subsequently, the rats were anesthetized with a combination of ketamine (40 mg/kg b. wt) and xylazine (5 mg/kg b. wt) and euthanized by cervical dislocation. Blood was drawn from the heart and collected in tubes, which were then centrifuged at 3000 g for 15 minutes to obtain serum for subsequent biochemical analysis. The pancreas and liver tissues were removed and subjected to histopathological examination. Another portion of the liver was homogenized to determine the activity of lipid peroxidation, antioxidant enzymes, and levels of certain proinflammatory cytokines.

**Biochemical analysis**

The levels of serum aspartate aminotransferase (AST), creatinine, alanine aminotransferase (ALT), uric acid, and urea, as well as triglycerides (TG), total lipids, high-density lipoprotein cholesterol (HDL-C), and total cholesterol (TC) were assessed using a commercial enzymatic kit from Bio-Diagnostic Co. in Egypt. Very low-density lipoprotein cholesterol (VLDL-C) and low-density lipoprotein cholesterol (LDL-C) levels were measured utilizing the methods described by Lee and Nieman [9] and Castelli et al. [10], respectively.

**Antioxidant, Glucose metabolism, and Inflammatory biomarkers**

Liver tissue samples were homogenized with phosphate buffer (pH7.4) at ice-cold temperatures and centrifuged at 3000 × g for 15 minutes to separate the supernatant from the homogenates, which were used for biochemical analysis. The activity levels of catalase (CAT), glutathione peroxidase (GPx), malondialdehyde (MDA), and superoxide dismutase (SOD) were measured in the liver homogenate using the methods described by Nishikimi et al. [11], Aebi [12], Ohkawa et al. [13], and Rotruck et al. [14], respectively.

The hepatic glycogen content, glucose-6-phosphatase activity, Insulin level, and glucokinase activity were evaluated utilizing the methods of Roe and Dailey [15] , Harper [16], Ojo et al.17 [17] and Brandstrup et al. [18], respectively, in the liver homogenate.

Additionally, the levels of pro-inflammatory cytokines, including interleukin-6 (IL-6) and tumor necrosis factor-α (TNF-α), were measured in the liver homogenate using a test reagent kit from CUSABIO Company, following the manufacturer's instructions.

**Histopathological examination:**

The pancreas and liver specimens were fixed using a formalin solution (10%) before being washed with water, dried out using increasing concentrations of alcohol, and purified with Xylene. Slices of paraffin (5 µm) were taken from the samples and treated with hematoxylin-eosin (HE) stain to perform a standard histopathological examination according to Bancroft and Gamble's approach [18].

**Statistical analysis**

The statistical analysis in this study involved the use of SPSS 21.0 software. One-way analysis of variance (ANOVA) was performed to compare the different groups, followed by Tukey's post hoc test to determine the smallest significant difference between the control group and the other animal groups. A significance level of p < 0.05 was used for all tests. The results for each group are presented as means ± standard errors (SE) based on the data collected from ten animals in each group.

**Results and discussion**

**Table S1. Total compounds of phenolic, total flavonoids, and Antioxidant activity of strawberry juice** *

| **Component** | **Strawberry juice** |
| --- | --- |
| **TPC (mg GAE/g)** | 22.03 ± 1.74 |
| **TFC (mg QE /g)** | 29.7 ± 2.89 |
| **DPPH (μmol TE/g)** | 25.22 ± 1.0 |
| **ABTS (μmol TE/g)** | 144.69 ± 13.44 |
| **FRAP (μmol TE/g)** | 47.59 ± 3.14 |

TE: Trolox equivalent, each value is expressed as the mean ± SE (n = 3).

**Table S2. Flavonoid and Phenolic compounds determined by HPLC in Strawberry juic**

| Components | RT (min) | Conc. (μg/mg) |
| --- | --- | --- |
|  | | |
|  | Phenolic compounds |  |
| Catechol | 4.0 | 10.22 |
| Syringenic | 5.0 | 8.22 |
| Cinnamic | 7.0 | 5.16 |
| Caffeic | 8.0 | 2.34 |
| Pyrogallol | 9.3 | 4.56 |
| Ferulic | 11.0 | 1.44 |
| Flavonoid compounds | | |
| Naringin | 4.6 | 2.14 |
| Rutin | 5.2 | 13.26 |
| Quercetin | 6.9 | 10.22 |
| Kaempferol | 8.1 | 1.46 |
| Luteolin | 9.0 | 4.06 |
| Apigenin | 10.0 | 5.11 |
| Catechin | 12.01 | 3.07 |

**Table S3. Histopathological lesion score in the liver**

| Histopathological lesion | NC | S | DC | DC+ S | DC+ Met |
| --- | --- | --- | --- | --- | --- |
| Hepatocellular vacuolar degeneration | 0,0,0,0,0 | 0,0,0,0,0 | 3,3,2,3,2 | 1,1,0,0,0 | 1,1,0,0,0 |
| Kupffer cells activation | 0,0,0,0,0 | 0,0,0,0,0 | 3,2,3,3,2 | 1,1,1,2,2 | 1,2,0,0,0 |
| Thickening in the wall of bile duct | 0,0,0,0,0 | 0,0,0,0,0 | 2,3,2,3,3 | 0,0,0,0,0 | 1,0,0,0,0 |
| Portal edema | 0,0,0,0,0 | 0,0,0,0,0 | 1,2,1,2,2 | 1,0,0,0,0 | 0,1,0,0,0 |

(0) indicated no changes, (1), (2) and (3) indicated mild, moderate and severe changes. **NC, normal control; S, strawberry juice; DC, diabetic control; Met, metformin**

**Table S4. Histopathological lesion score in the pancreas**

| Histopathological lesion | NC | S | DC | DC+ S | DC+ Met |
| --- | --- | --- | --- | --- | --- |
| Congestion | 0,0,0,0,0 | 0,0,0,0,0 | 1,1,2,2,1 | 1,1,0,0,0 | 1,0,0,0,0 |
| Vacuolation of cells of islet’s of Langerhan’s | 0,0,0,0,0 | 0,0,0,0,0 | 3,3,2,2,2 | 0,0,0,0,0 | 0,1,1,1,0 |
| Necrosis of cells of islet’s of Langerhan’s | 0,0,0,0,0 | 0,0,0,0,0 | 3,3,2,2,2 | 0,0,0,0,0 | 0,0,0,0,0 |
| Hyperplasia and cystic dilatation of pancreatic duct | 0,0,0,0,0 | 0,0,0,0,0 | 3,3,3,3,3 | 0,0,0,0,0 | 1,0,0,0,0 |

(0) indicated no changes, (1), (2) and (3) indicated mild, moderate and severe changes. **NC, normal control; S, strawberry juice; DC, diabetic control; Met, metformin**


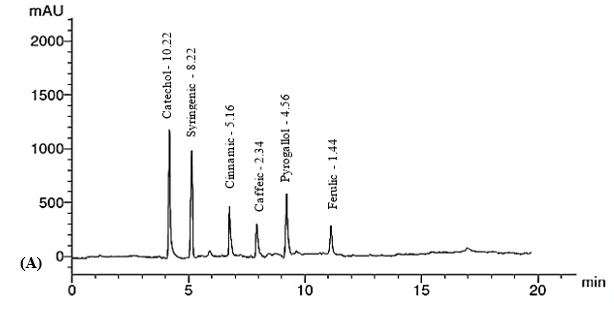


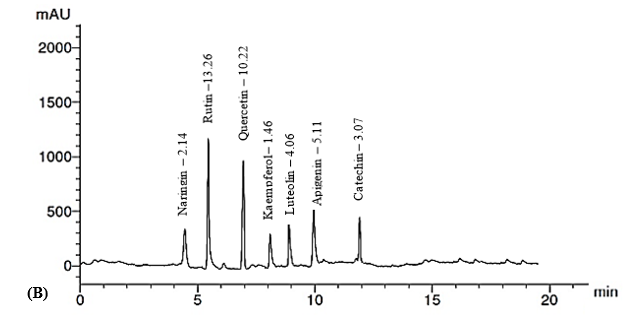


**Figure S1. HPLC chromatograms of Strawberry juice phenolic acids (A) and flavonoids (B)**

| 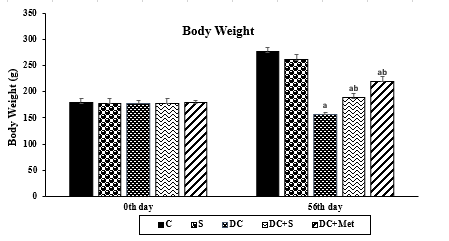 | |
| --- | --- |
| 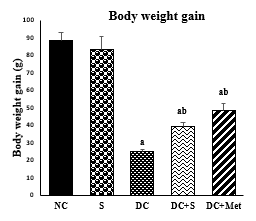 | 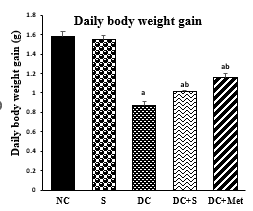 |

**Figure S2: Effects of Strawberry Juice on body weight (means ± SE). a and b are significant at p < 0.05 in comparison of groups with normal and diabetic control groups, respectively. NC, normal control; S, strawberry juice; DC, diabetic control; Met, metformin**

| 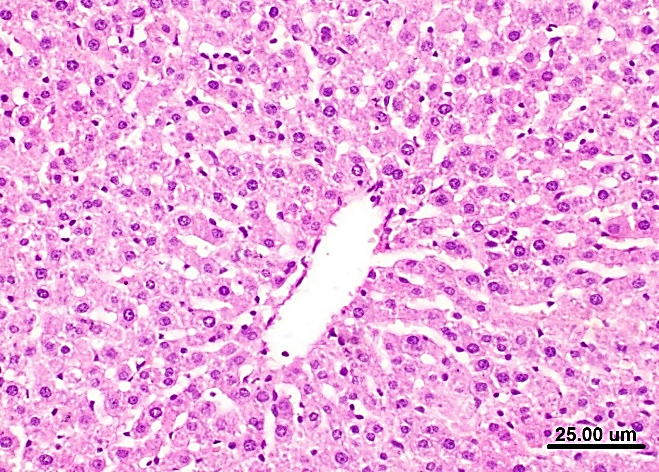 | 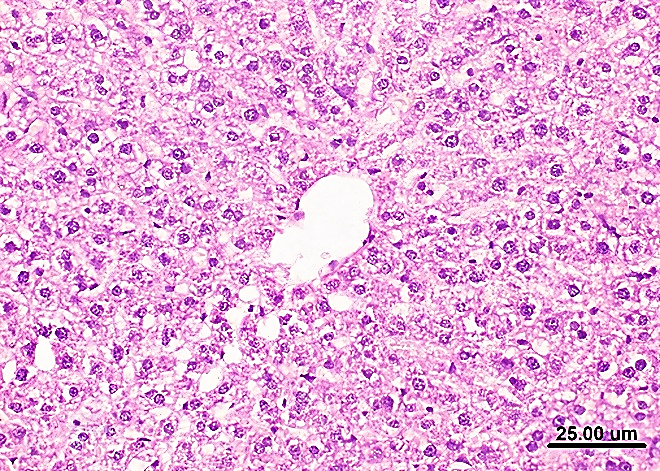 |
| --- | --- |
| **Figure (A): Photomicrograph of liver of rat from NC group displaying no histopathological lesions (H & E X 400, scale bar 25μm)** | **Figure (B): Photomicrograph of liver of rat from S group displaying no histopathological lesions (H & E X 400, scale bar 25μm).** |
| 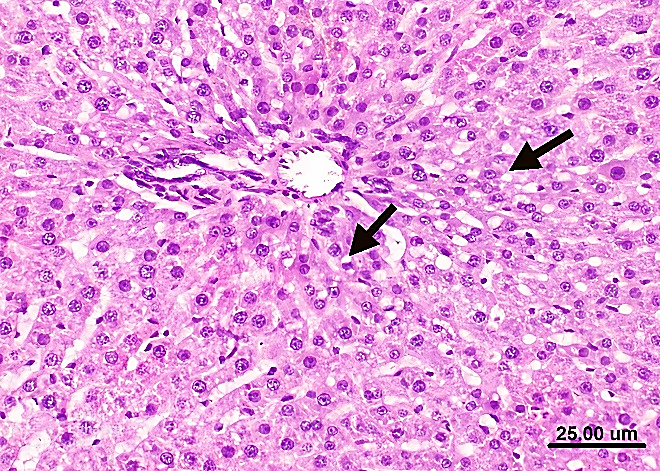 | 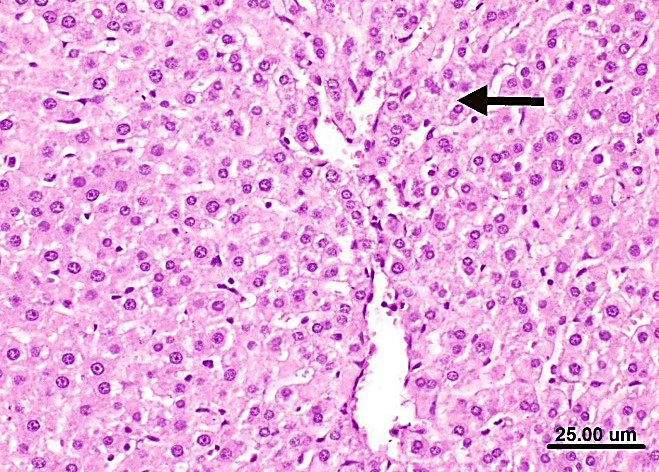 |
| **Figure (C): Photomicrograph of liver of rat from DC group displaying hepatocellular vacuolar degeneration (H & E X 400, scale bar 25μm).** | **Figure (D): Photomicrograph of liver of rat from DC+S group displaying slight vacuolation of some hepatocytes (H & E X 400, scale bar 25μm).** |
| 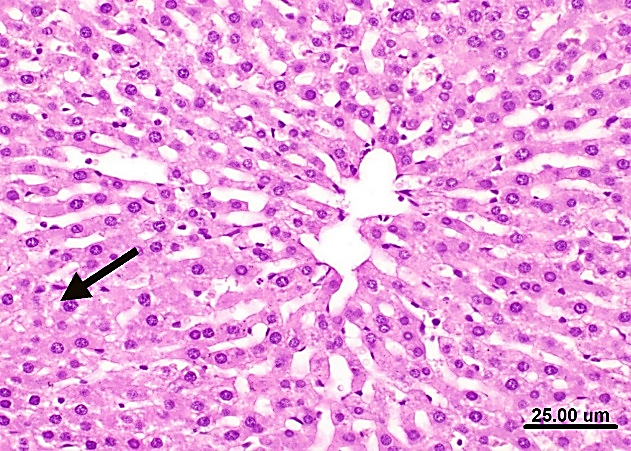 |  |
| **Figure (E): Photomicrograph of liver of rat from DC+Met group displaying slight vacuolation of some hepatocytes (H & E X 400, scale bar 25μm).** |  |

**Figure S3: Histopathological examination of liver. NC, normal control; S, strawberry juice; DC, diabetic control; Met, metformin**

| 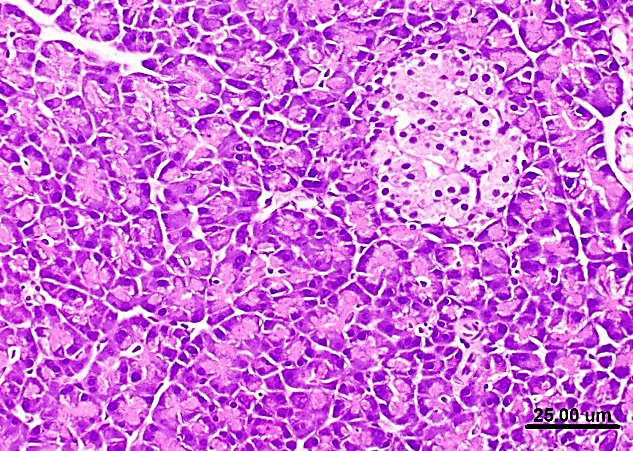 | 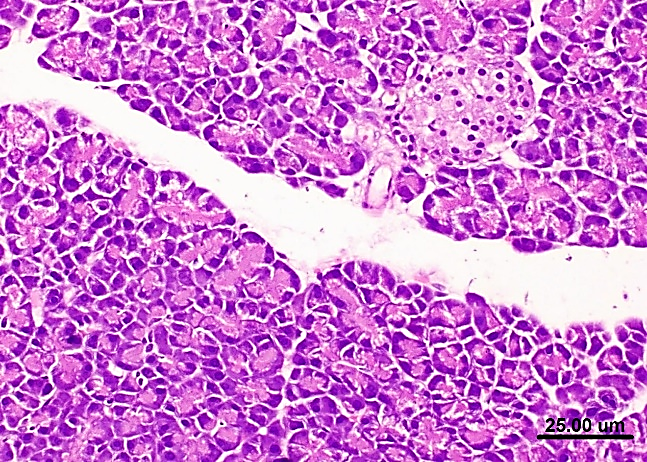 |
| --- | --- |
| **Figure (A): Photomicrograph of pancreas of rat from NC group displaying normal islets of Langerhans and normal pancreatic acini (H & E X 400, scale bar 25μm).** | **Figure (B): Photomicrograph of pancreas of rat from S group displaying normal pancreatic acini and normal islets of Langerhan’s (H & E X 400, scale bar 25μm).** |
| 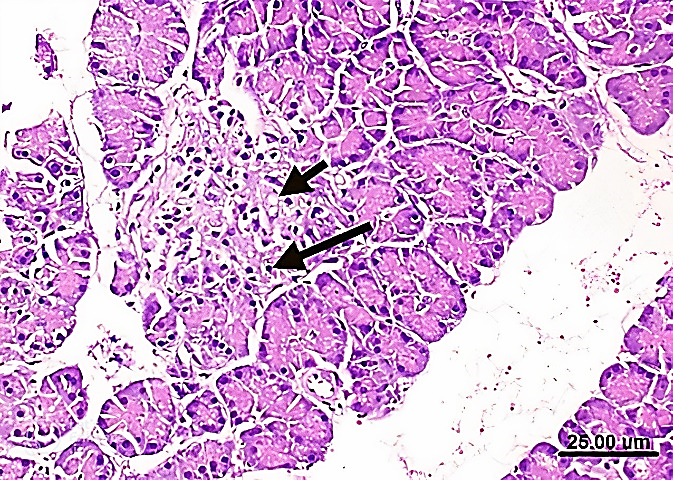 | 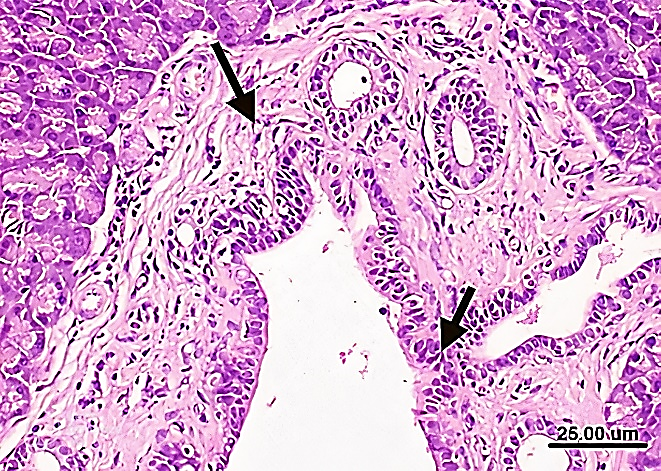 |
| **Figure (C): Photomicrograph of pancreas of rat from DC group displaying marked vacuolation and necrosis of islets of Langerhans cells (H & E X 400, scale bar 25μm).** | **Fig. (D): Photomicrograph of pancreas of rat from DC group showing marked hyperplasia of epithelial lining pancreatic duct and thickening of its wall (H & E X 400, scale bar 25μm).** |
| 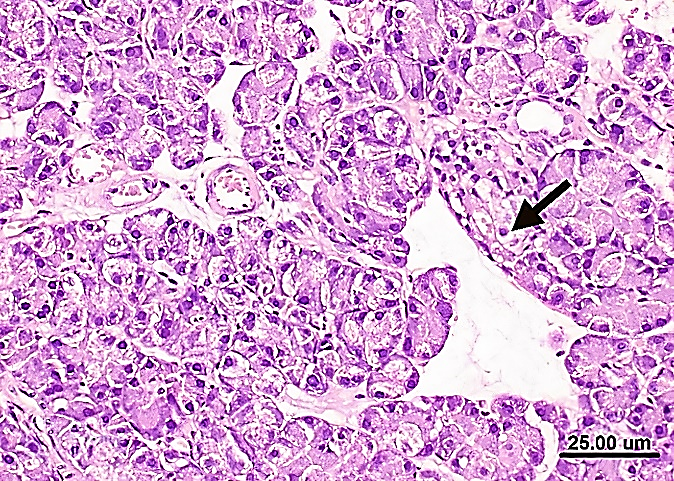 | 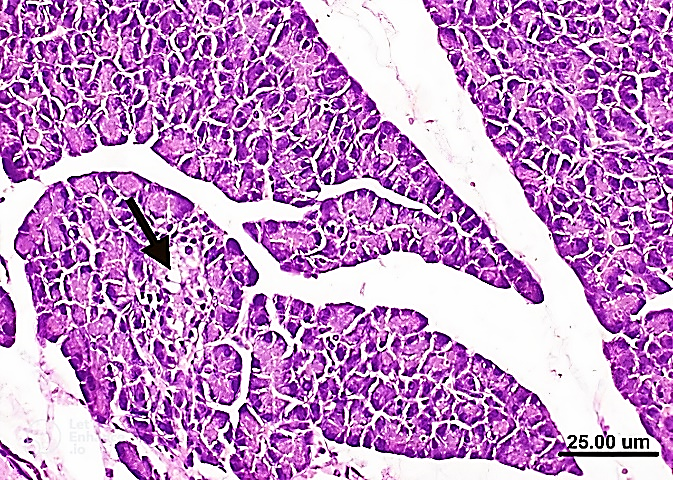 |
| **Figure (E): Photomicrograph of pancreas of rat from DC+S group showing vacuolation of some cells of islets of Langerhan’s (H & E X 400, scale bar 25μm).** | **Figure (F): Photomicrograph of pancreas of rat from DC+Met group displaying vacuolation of some cells of islets of Langerhan’s (H & E X 400, scale bar 25μm).** |

**Figure S4: Histopathological examination of pancreas. NC, normal control; S, strawberry juice; DC, diabetic control; Met, metformin**

**Reference List**

1. Meziti A, Bouriche H, Meziti H, Kadaa S, Abderrahmane S
   (2017) Antioxidant and anti-inflammatory activities of
   Rubus fruticosus and Zizyphus vulgaris methanol extracts.
   Int J Pharm Pharm Sci 9: 69–76.
2. Benzie IFF, Strain JJ (1996) The ferric reducing ability of plasma (FRAP) as a measure of “antioxidant power”: the FRAP assay. Anal Biochem 239: 70–76.
3. Saija A, Tomaino A, Lo Cascio R, Rapisarda P, Dederen JC (1998) *In vitro* antioxidant activity and *in vivo* photoprotective effect of red orange extract. Int J Cosmet Sci 20: 331–342
4. Kutlu T, TakimK, Çeken B, Kizil M (2014) DNA damage protecting activity and in vitro antioxidant potential of the methanol extract of Cherry (Prunus avium L). J Med Plants Res 8: 715–726.
5. Gazwi HSS, Shoeib NA, Mahmoud ME, Soltan OIA, Hamed MM, Ragab AE (2022) Phytochemical Profile of the Ethanol Extract of Malvaviscus arboreus Red Flower and Investigation of the Antioxidant, Antimicrobial, and Cytotoxic Activities. Antibiotics. 11(11):1652. https://doi.org/10.3390/antibiotics11111652
6. Samarghandian S, Azimi-Nezhad M, Farkhondeh T( 2017) Catechin treatment ameliorates diabetes and its complications in streptozotocin-induced diabetic rats. Dose-Response 15: 1–7.
7. Putri MD (2021) The Effect of Strawberry Juice on Homa-IR Level in Rat Model of Type 2 Diabetes Mellitus.Int J Nutr Sci 6(3):134-140
8. Dimo T, Rakotonirina S V, Tan PV, Azay J, Dongo E, Kamtchouing P, Gros G (2007) Effect of Sclerocarya birrea (Anacardia ceae) Stem bark methylene chloride/methanol extract on streptozotocin-diabetic rats. Journal of Ethnopharmacology110:434-438
9. Lee WY, Nieman TA (1996) Effect of organic solvent on tris (2, 2′-bipyridyl) ruthenium (III) chemiluminescent reactions in flowing streams. *Analytica chimica acta* *334*(1-2): 183-191.
10. Castelli, W P, Doyle J T, Gordon T, Hames C G, Hjortland M C, Hulley S B, Zukel W J (1977) HDL cholesterol and other lipids in coronary heart disease. The cooperative lipoprotein phenotyping study. Circulation 55(5): 767-772.
11. Nishikimi M, Rao N A, Yagi K (1972) The occurrence of superoxide anion in the reaction of reduced phenazine methosulfate and molecular oxygen Biochemical and biophysical research communications 46(2): 849-854.
12. Aebi H (1984) Catalase in vitro. Methods Enzymol. Academic Press. 105: 121-126.
13. Ohkawa H, Ohishi W, Yagi K (1979) Assay for lipid peroxides in animal tissues by thiobarbituric acid reaction. Analytical Biochem 95(2): 351-358.
14. Rotruck JT, Pope AL, Ganther HE, Swanson AB, Hafeman DG, Hoekstra W (1973)Selenium: biochemical role as a component of glutathione peroxidase. Sci, 179(4073): 588-590.
15. Roe JH, Dailey RE (1966) Determination of glycogen with the anthrone reagent. Analytical biochem 15: 245-250.
16. Harper AE (1959) Hormonal factors affecting glucose 6-phosphatase activity. 2. Some effects of diet and of alloxan diabetes in the rat. Biochemical J, 71(4): 702.
17. Ojo OA, Ajiboye BO, Ojo AB, Oyinloye BE, Imiere OD, Adeyonu O (2017) Ameliorative potential of *Blighia sapida* K.D. Koenig bark against pancreatic beta-cell dysfunction in alloxan-induced diabetic rats. J Complement Integr Med , 17:14(3) <https://doi.org/10.1515/jcim-2016-0145>.
18. Brandstrup N, Kirk J.E, Bruni C (1957) The hexokinase and phosphoglucoisomerase activities of aortic and pulmonary artery tissue in individuals of various ages. J. of Gerontology12(2):166-171.
19. Bancroft JD, Gamble M (2008) Theory and practice of histological techniques 7th ed, Churchill Livingstone London, UK,; pp. 125–138 and 328–329.
